# Proteomic analysis reveals that aging rabbit vocal folds are more vulnerable to changes caused by systemic dehydration

**DOI:** 10.1186/s12864-022-08975-x

**Published:** 2022-11-21

**Authors:** Naila C. do Nascimento, Taylor W. Bailey, Andrea P. Santos, Chenwei Duan, Rodrigo Mohallem, Jackeline Franco, Uma K. Aryal, Jun Xie, Abigail Cox, M. Preeti Sivasankar

**Affiliations:** 1 Department of Speech, Language, and Hearing Sciences, Purdue University, West Lafayette, Indiana, 47907 USA; 2 Department of Comparative Pathobiology, Purdue University, West Lafayette, Indiana, 47907 USA; 3 Weldon School of Biomedical Engineering, Purdue University, West Lafayette, Indiana, 47907 USA; 4 Purdue Proteomics Facility, Bindley Bioscience Center, Discovery Park, Purdue University, West Lafayette, Indiana, 47907 USA; 5 Department of Statistics, Purdue University, West Lafayette, Indiana, 47907 USA

**Keywords:** Young, Aging, Larynx, Vocal folds, Water restriction, Dehydration, Proteome

## Abstract

**Background:**

Older adults are more prone to develop systemic dehydration. Systemic dehydration has implications for vocal fold biology by affecting gene and protein expression. The objective of this study was to quantify vocal fold protein changes between two age groups and hydration status, and to investigate the interaction of age and hydration status on protein expression, which has not been investigated in the context of vocal folds before. Comparative proteomics was used to analyze the vocal fold proteome of 6.5-month-old and > 3-year-old rabbits subjected to water ad libitum or water volume restriction protocol.

**Results:**

Young and older adult rabbits (*n* = 22) were either euhydrated (water ad libitum) or dehydrated by water volume restriction. Dehydration was confirmed by body weight loss of − 5.4% and − 4.6% in young and older groups, respectively, and a 1.7-fold increase of kidney *renin* gene expression in the young rabbits. LC-MS/MS identified 2286 proteins in the rabbit vocal folds of young and older adult rabbits combined. Of these, 177, 169, and 81 proteins were significantly (*p* ≤ 0.05) affected by age, hydration status, or the interaction of both factors, respectively. Analysis of the interaction effect revealed 32 proteins with opposite change patterns after dehydration between older and young rabbit vocal folds, while 31 proteins were differentially regulated only in the older adult rabbits and ten only in the young rabbits in response to systemic dehydration. The magnitude of changes for either up or downregulated proteins was higher in the older rabbits. These proteins are predominantly related to structural components of the extracellular matrix and muscle layer, suggesting a disturbance in the viscoelastic properties of aging vocal fold tissue, especially when subjected to systemic dehydration.

**Conclusions:**

Water restriction is a laboratory protocol to assess systemic dehydration-related changes in the vocal fold tissue that is translatable to human subjects. Our findings showed a higher number of proteins differentially regulated with a greater magnitude of change in the vocal folds of older adult rabbits in the presence of systemic dehydration compared to younger rabbits. The association of these proteins with vocal fold structure and biomechanical properties suggests that older human subjects may be more vulnerable to the effects of systemic dehydration on vocal function. The clinical implications of these protein changes warrant more investigation, but age should be taken into consideration when evaluating vocal treatment recommendations that interfere with body fluid balance.

**Supplementary Information:**

The online version contains supplementary material available at 10.1186/s12864-022-08975-x.

## Background

Vocal folds are specialized viscoelastic tissue located in the larynx responsible for vibrating the airstream to produce sound. The larynx and vocal folds age as people age, resulting in physiological speech and voice changes [1–3]. The process of physiological voice aging is referred to as presbyphonia [4]. Several studies have described the changes associated with this process [2, 3, 5–7]. Generally, age-related alterations are attributed to the anatomy and physiology of the structural support of vocal folds, such as muscle and vocal fold atrophy, stiffness of the collagen layer, thinning of elastin fibers, and decreased mucous production [3, 4, 8, 9]. These alterations may result in poor vocal projection, reduced loudness, hoarseness, change in pitch, decreased phonation time, tremor, breathiness, and decreased accuracy of motor control [3, 4, 6, 8, 10]. Understanding the effects of aging on the systems that support voice and speech is critical to discriminating normal physiology from pathology and allows for studies focused on the effects of concomitant aggravating factors on voice health, such as dehydration, a common condition in older individuals [11, 12].

Maintaining water homeostasis is essential to preserve life, as virtually all biological and chemical reactions in the body require an aqueous medium [13–15]. Additionally, water serves as a carrier of nutrients required to maintain viable cells, eliminates toxic compounds from the body, and provides structural support for tissues [14, 15]. Thus, water and electrolyte balance is tightly regulated [14]. Total body water decreases with age [14, 16, 17]; in humans, the water body changes range from 75% in infants to 55% in the elderly [13] and 45% in older women, making older individuals more prone to developing dehydration [14]. There are multiple factors contributing to the development of dehydration as we age [13, 14], including the increased proportion of body fat over lean body mass [14, 17], decreased renal function impairing efficient water conservation [14, 17], decreased sense of thirst [18], and the presence of comorbidities [14, 19]. Although several studies have focused on fluid homeostasis, dehydration, and contributing factors in the aging process, the impact of systemic dehydration on older subjects in the context of vocal fold biology has not yet been investigated.

Vocal fold hydration is a typical recommendation to maintain vocal health; however, many aspects of the underlying mechanisms of its benefits remain unclear. Studies focused on systemic (de)hydration – manipulating water consumption and cellular absorption – in humans have shown that dehydration negatively affects vocal fold physiology and function. However, due to ethical concerns, the use of human subjects is limited to mechanistic studies of laryngeal imaging, acoustic assessments and aerodynamic measures of voice, or postmortem studies [20–24]. In addition, some human studies combined systemic and surface dehydration as challenges, making it difficult to determine the specific biological effects of systemic (de)hydration in the vocal folds [21, 22, 25]. Nevertheless, these studies suggest that dehydration negatively impacts voice production by altering the viscoelasticity properties of the vocal folds. Conversely, animal models allow for ex vivo and in vivo research, using different dehydration protocols. Studies using excised canine larynges have shown that osmotically induced dehydration increases vocal fold stiffness and viscosity [26] and that changes in viscosity affect the vocal fold vibration [27]. Moreover, a decrease in hyaluronan content in the lamina propria was observed in vocal folds from water-restricted rats [28]; hyaluronan is a critical component for maintaining hydration and optimal tissue viscosity [24]. These results support the premise that hydration status alters vocal fold viscoelasticity, as observed in human studies.

In addition to clinical and experimental studies, research focused on the underlying molecular mechanisms of hydration has shown that systemic dehydration affected gene and protein expression in the vocal folds of different animal models [28–30]. Transcriptome studies of the vocal folds from furosemide-induced systemically dehydrated rabbits revealed hundreds of differentially expressed genes compared with euhydrated rabbits [29]. In that study, epithelial and cell adhesion-related genes were downregulated, suggesting systemic dehydration may perturb the vocal fold epithelium. Proteomic studies using the same rabbit model revealed dozens of differentially regulated proteins and suggested novel molecular evidence for increased susceptibility to oxidative stress [30] as observed in rats after acute vocal fold injury [31, 32]. Interestingly, treatment with antioxidants prevented age-associated changes in rat vocal folds [9]. Collectively, these findings represent strong evidence that systemic dehydration adversely affects vocal fold biology and that systemic hydration is an effective practice to improve voice quality; however, the interaction between hydration status and aging is an unexplored topic in voice research.

To start filling this gap, our study aimed to identify protein signatures and related mechanisms associated with the intersection of age and hydration status in vocal fold biology. We used comparative label-free quantitative proteomics to analyze the alterations of protein expression patterns in the vocal folds of 6-month-old and > 3-year-old rabbits in response to water ad libitum or water restriction-dehydration protocols. Proteomics-based technology was utilized given its high sensitivity and high-throughput capacity [33]. We used the rabbit as in vivo model given the similarities to human vocal fold anatomy and biology [34, 35]. Furthermore, the rabbit is a well-validated model in studies focused on the vocal folds [29, 30, 36–45]. Water volume restriction was chosen as the systemic dehydration method to mimic a more physiologically-realistic state that can be better translated to human behavior [46–48]. Our comparative proteomics analysis showed a higher number of proteins, related to tissue structure and viscoelastic properties, differentially regulated and with a greater magnitude of change in the vocal folds of older adult rabbits due to systemic dehydration compared to younger rabbits, suggesting a higher vulnerability of aging vocal folds to systemic dehydration.

## Results

### Water intake

**Fig. 1:**
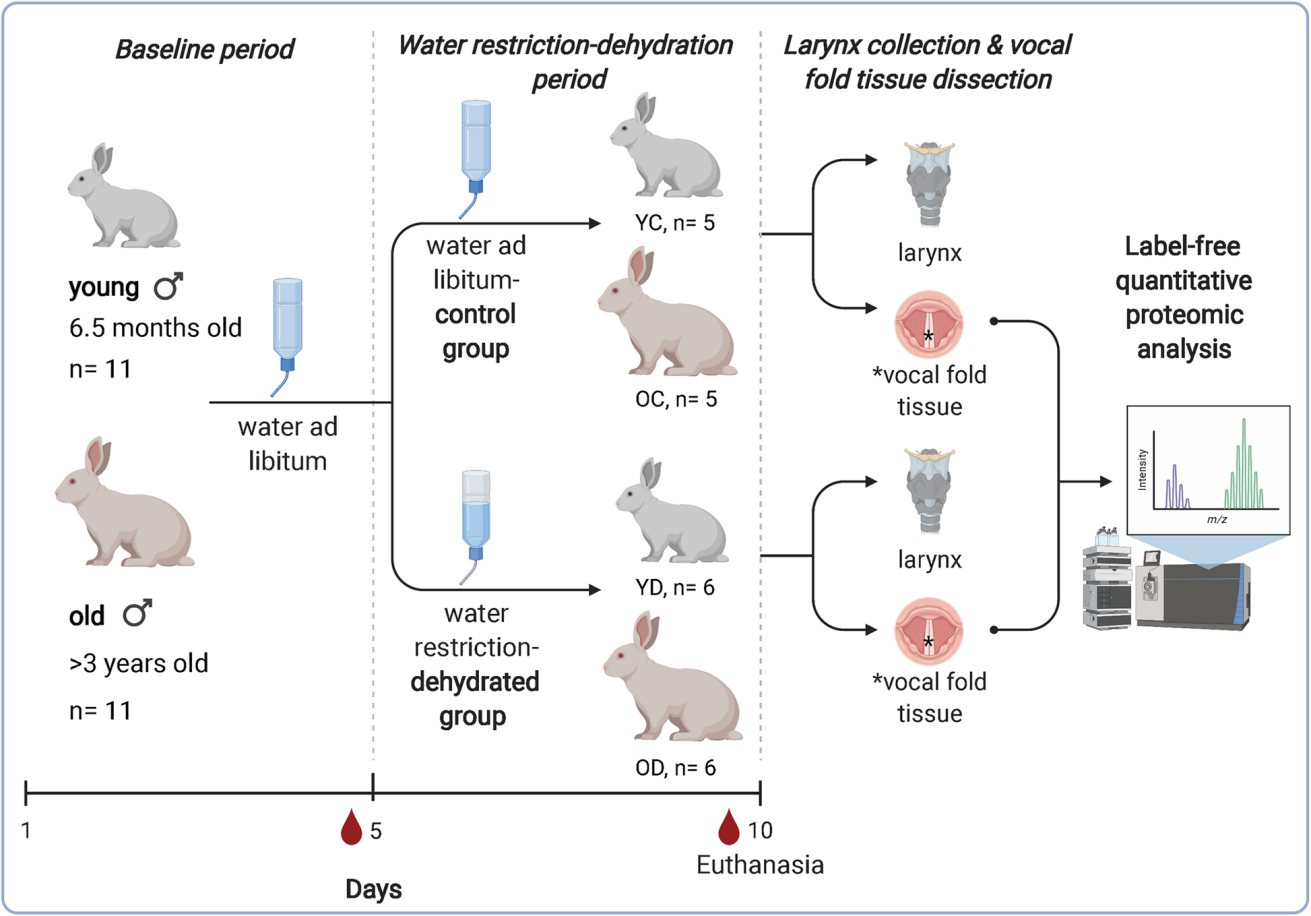
Experimental timeline and water restriction-dehydration protocol. The young (6.5 months old; n = 11) and older adult (3.2–3.7 years old; n = 11) male rabbits were acclimatized for one week. Then, rabbits were assigned to control (water ad libitum; n = 5 young, and n = 5 older adults) or dehydrated (water volume restriction; n = 6 young, and n = 6 older adults) groups. The experiment had 10 days: a *baseline period* of 5 days, where all animals received water ad libitum, and the *water restriction-dehydration period* of 5 days, where the dehydrated group had the water volume restricted to half of their average baseline consumption. Blood was collected on the last day of the *baseline period* (pre-dehydration) and at the end of dehydration for packed cell volume and total solids evaluation. At the end of the *dehydration period*, rabbits were euthanized and vocal fold tissue was collected for proteomic and RT-qPCR analyses. The figure was created with BioRender.com

**Fig. 2:**
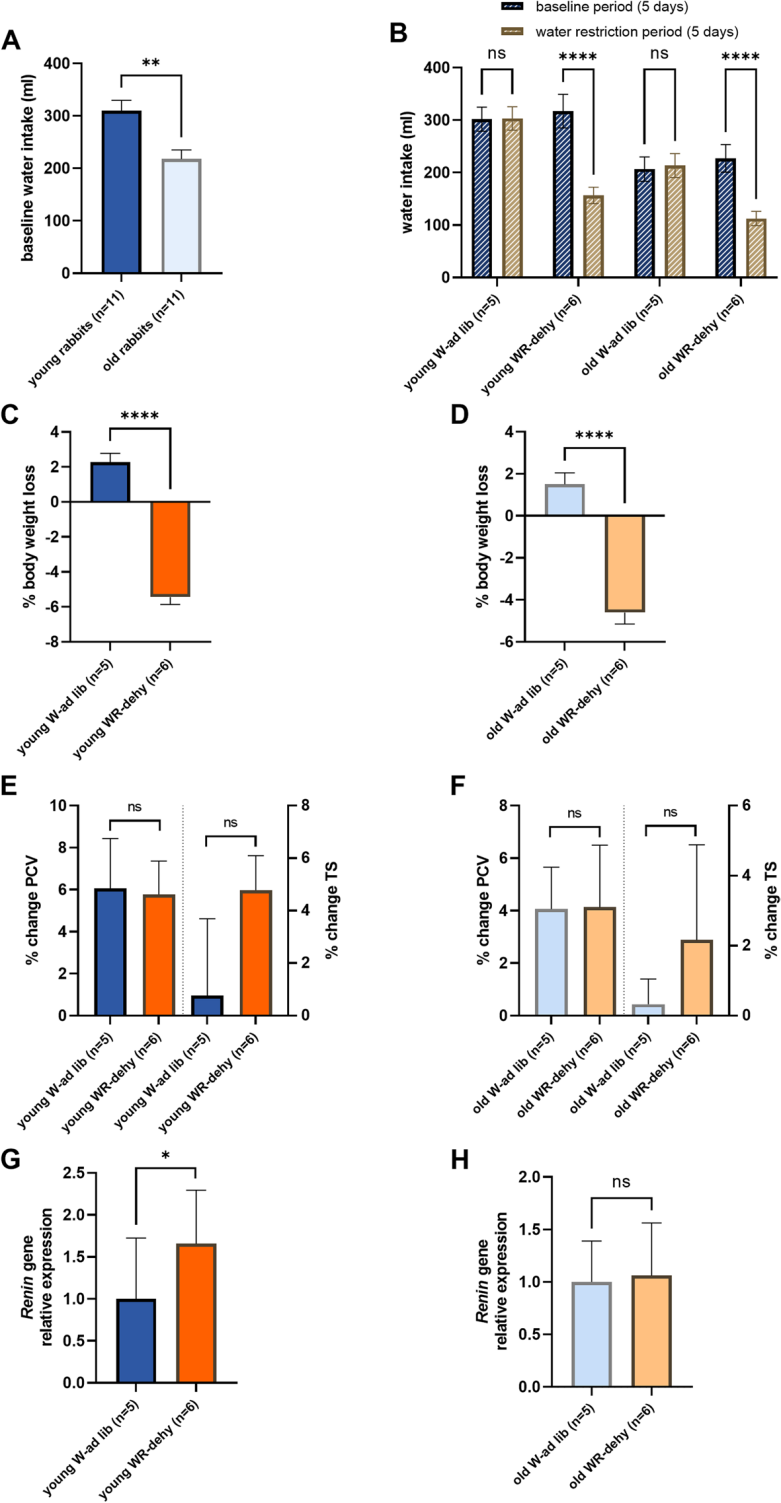
Water intake and dehydration markers. **a** Water intake by young and older adult rabbits during the baseline period where animals were given water ad libitum. Older adult rabbits consumed ~ 30% less water than young rabbits. **b** Comparison of water intake by young and older adult rabbits between the control and dehydrated groups during the experiment’s baseline and water restriction periods. During the water restriction period, both young and older adult rabbits in the control group received water ad libitum, while those in the dehydrated group received a restricted volume of water. **c, d** Percentage body weight loss. Both young and older adult rabbits in the dehydrated group had an average body weight loss of − 5.4% and − 4.6%, respectively, at the end of the water restriction period compared to the control rabbits. **e, f** Percentage change in packed cell volume (PCV) and total solids (TS) after the water restriction period. Water restriction did not alter the PCV or TS of rabbits in the dehydrated group in either young or older adult rabbits. **g, h** Kidney *renin* gene expression. The expression level of *renin* was increased in the young water restriction-dehydrated rabbits but not in the older-dehydrated rabbits compared to their respective controls. W-ad lib: control groups that received water ad libitum throughout the study. WR-dehy: dehydrated groups that received water restricted volume during the water restriction period of the study. All bars show average values ± SEM. *p ≤ 0.05, **p ≤ 0.01, ****p ≤ 0.0001. ns: non-significant

The water intake protocol was divided into two stages: *baseline* (5 days, day 1 to 5) and *water restriction-dehydration* period (5 days, day 6 to 10) (Fig. 1). The water intake of euhydrated-control rabbits (young and older adults), which received water ad libitum during the entire experiment, did not change throughout the study, although the baseline water intake was 30% lower for older rabbits than younger rabbits (Fig. 2a). For animals in the dehydrated group, water volume intake was reduced by 50% during the water restriction period compared to the volume consumed in the baseline period, as our water volume restriction protocol intended (Fig. 2b).

### Systemic dehydration markers

The body weights of all rabbits were monitored daily during the entire experiment as a marker of systemic dehydration. Systemic dehydration was verified at the end of the *water restriction period* by body weight loss of − 5.4% in the young-dehydrated rabbits and − 4.6% in the older-dehydrated group, while young and older-control rabbits gained 2.2 and 1.5% body weight, respectively (Fig. 2c–d). The percentage changes in packed cell volume (PCV) and total solids (TS), from baseline to the end of the dehydration period, were compared between control and dehydrated rabbits in both age groups as blood markers of dehydration. PCV and TS did not change significantly between control and dehydrated rabbits in either of the age groups (Fig. 2e–f). Finally, at the end of the experiment, renal *renin* gene expression was compared between controls and dehydrated rabbits in the young and older adult groups. Renin expression is expected to increase due to systemic dehydration, which was verified for young rabbits (1.7-fold increase) but not for the older adult rabbits compared to their respective controls (Fig. 2g–h).

### Comparative proteomics of vocal folds of young and old rabbits under euhydration (control condition) or water restriction-dehydration

Label-free quantitative (LFQ) proteomics was used to determine the proteome changes related to age, hydration status, and interaction between age and hydration in rabbit vocal folds. Proteins identified at 1% FDR, with at least one unique peptide and MS/MS counts ≥2, were considered true identifications. These data were uploaded to the Perseus platform, and after removal of “contaminants”, “reverse”, and “only identified by site”, a total of 3667 proteins were identified. Then, the data were Log2 transformed and filtered to keep only proteins with a minimum of three valid LFQ (non-zero) values in at least one of the four groups: young-control (YC), young-dehydrated (YD), older-control (OC), or older-dehydrated (OD) rabbits. After filtering and imputation of values based on normal distribution, 2286 proteins were identified and used in further analyses. Two-way ANOVA identified 177, 169, and 81 proteins significantly (*p* ≤ 0.05) affected by age, hydration status, or interaction of both factors, respectively (Additional file 1: Table S1). Principal component analysis (PCA) was conducted in parallel with ANOVA to further characterize the collective contribution of proteins to the factors investigated. We considered the correlation of proteins affected by age and hydration status to principal component 1 (PC1) as an alternative to adjusted statistical significance as a criterion in defining proteins of interest (Additional file 2: Tables S2). These subsets of proteins with *p* ≤ 0.05 were then used for heat maps to show protein expression patterns across groups, and pathway and process enrichment analysis to define functional clusters. Analysis of the set of proteins that showed a significant interaction effect was performed to identify the pattern of changes in young and older rabbits’ vocal folds due to systemic dehydration. Results from these analyses are described below.

**Fig. 3:**
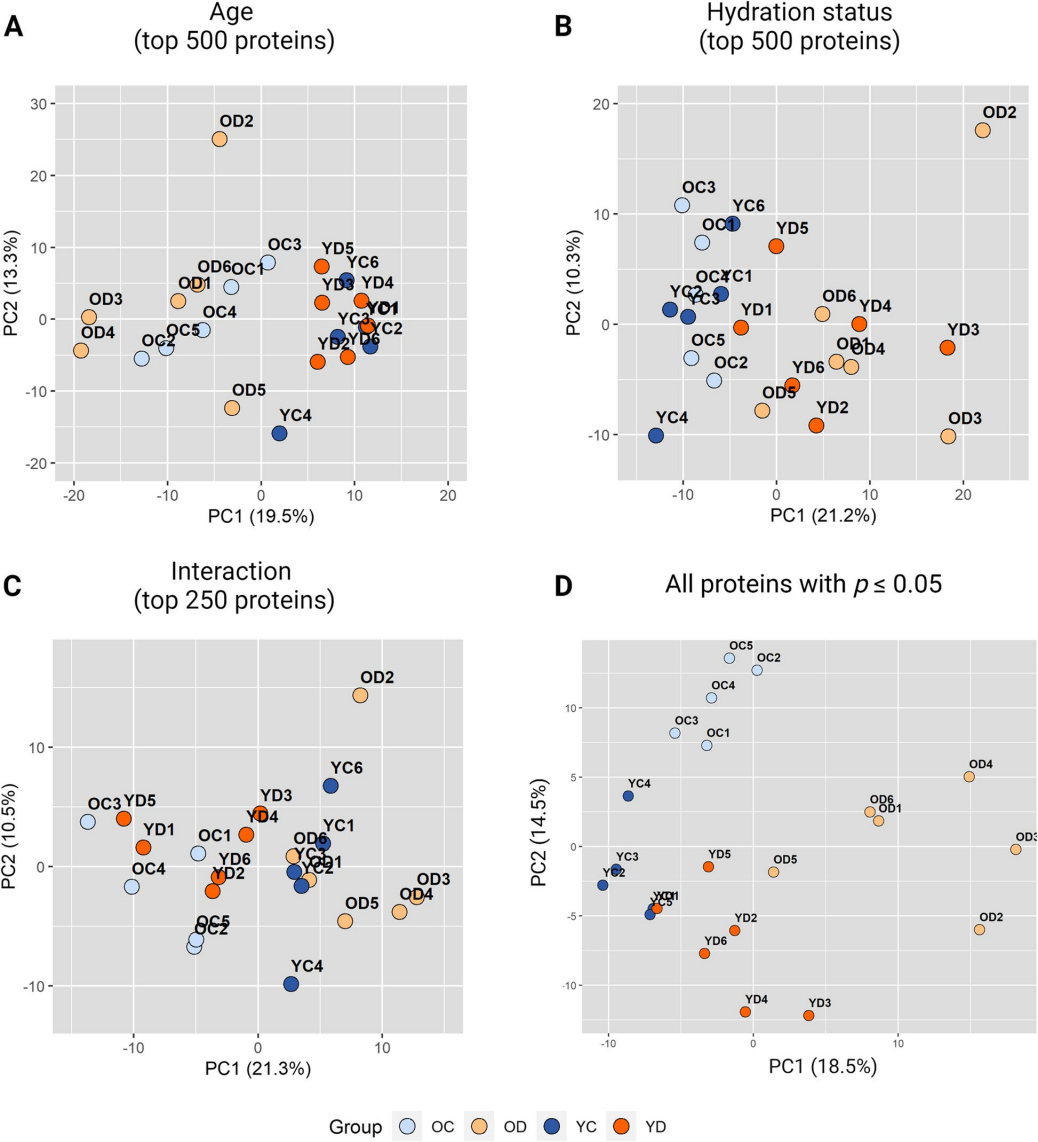
Principal component analysis (PCA) based on the proteomic data showing the separation of groups by **a** age, **b** hydration status, **c** interaction of both factors, and **d** all significant proteins from each effect combined. PCA plots are based on the top 500 proteins for age and hydration status main factors, top 250 proteins for interaction, and all significant proteins (*p* ≤ 0.05) organized by ascending *p*-value. OC: older-control; OD: older-dehydrated; YC: young-control; YD: young-dehydrated

Principal component analysis (PCA) based on the vocal fold proteome was performed for each main effect of age and hydration status, and interaction effect. Different subsets of proteins representing the top 500 (for age and hydration status), 250 (for interaction), and all significant proteins combined (*p* ≤ 0.05) arranged by ascending *p*-values for the respective factor were used to identify the separation between groups and the overall variance collectively provided by these proteins. Young (YC, YD) and older (OC, OD) age groups had a linear separation with the top 500 proteins (Fig. 3a), explaining 32.8% of the overall variance (PC1 and PC2 combined). The separation between hydration status groups (control: YC, OC vs. dehydrated: YD, OD) was observed for the top 500 proteins providing an overall explained variance of 31.5% (Fig. 3b). The distinction of groups considering the interaction effect was observed with the top 250 proteins explaining an overall variance of 31.8% (Fig. 3c). Finally, all significant proteins (*p* ≤ 0.05) identified for age, hydration status, and interaction effects provided an overall explained variance of 33%, with clear differentiation between the four groups and a more pronounced distinction of the older-dehydrated from the other groups (Fig. 3d). The focus of this study are the proteins with *p* ≤ 0.05 affected by interaction and those affected by age and hydration status that showed moderate to high correlation (≥ 0.6) with the respective first principal component indicating their contribution to the separation of biological groups (Additional file 2: Tables S2).

**Fig. 4:**
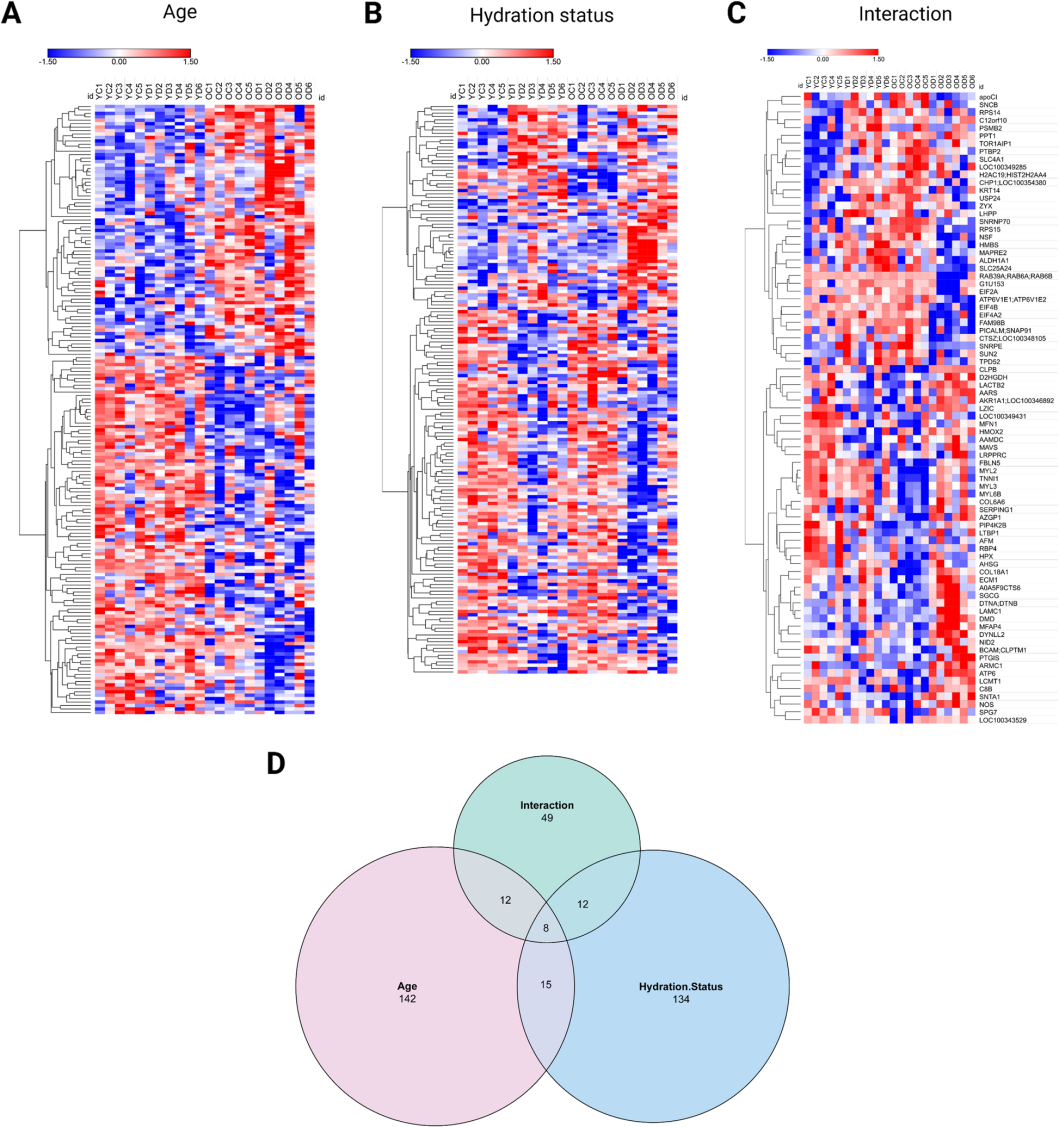
Heat maps of significant proteins identified by **a** age, **b** hydration status, or **c** interaction effect following Two-way ANOVA showing the patterns of protein expression in each group. **d** Venn diagram of significant proteins (*p* ≤ 0.05) based on the Two-way ANOVA results. Heat maps were created with Morpheus software (https://software.broadinstitute.org/morpheus). OC: older-control; OD: older-dehydrated; YC: young-control; YD: young-dehydrated

Hierarchical clustering of the significant proteins (*p* ≤ 0.05) into heat maps shows the pattern of up or downregulation of each protein subset by age, hydration status, and interaction (Fig. 4a–c). It is noteworthy that proteins affected by interaction show opposite expression patterns between young and older adult rabbits in response to dehydration (YD x OD) (Fig. 4c). A Venn diagram shows the overlap between the significant proteins affected by each individual factor and by the interaction (Fig. 4d). The majority of proteins are unique to each subset: age (142 proteins), hydration status (134 proteins), and interaction (49 proteins). Twenty-three common proteins were significantly affected by both main factors of age and hydration status. Twenty common proteins were significantly affected by age and interaction, and 20 were commonly found differentially regulated by hydration status and interaction. Only eight proteins were commonly found across the three subsets. The sets of unique proteins affected by only one factor or by the interaction highlights changes in protein expression patterns that are age- or hydration-dependent, with 49 proteins differentially affected in young and older adult rabbit vocal folds depending on the hydration level.

### Enrichment analysis for biological pathways and processes of significant proteins affected by age, hydration status, or interaction

**Fig. 5:**
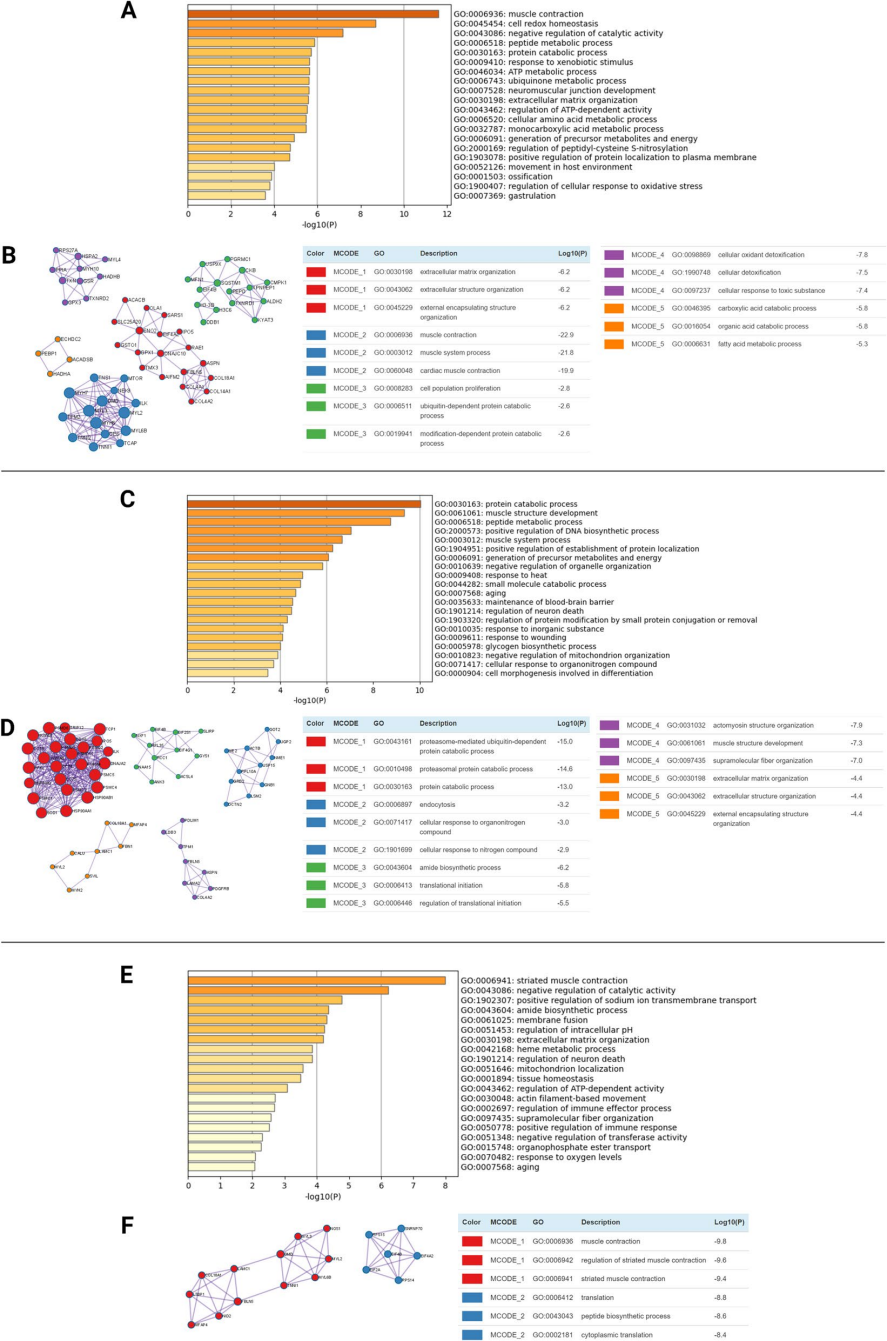
Enrichment gene ontology analysis and MCODE networks based on the protein-protein interaction (PPI) enrichment analysis. **a,c,e** Top 20 enriched terms identified based on the individual gene lists correspondent to significant proteins affected by age, hydration status or interaction, respectively. **b,d,f** PPI clusters identified by MCODE for individual gene lists based on the significant proteins identified by age, hydration status or interaction effects, respectively. Pathway and process enrichment analysis was applied to each MCODE cluster independently, and the three best-scoring terms by *p*-value are retained as the functional description of the corresponding clusters, shown in the tables adjacent to corresponding network plots. Nodes represent proteins, and edges represent the interaction between those proteins. Figures were obtained from enrichment and MCODE PPI analyses using Metascape

Enrichment analysis was performed using Metascape [49], and enriched terms were based on Gene Ontology Biological Processes (BP). The top 20 enriched terms for age, hydration status, and interaction are shown in Fig. 5a, c, and e, respectively. The Molecular Complex Detection (MCODE) was applied to identify functional clusters based on protein-protein interaction networks (Fig. 5b,d,f). Age-related top enrichment BP terms are associated with “muscle contraction”, “cell redox homeostasis”, “protein degradation”, and “extracellular matrix organization”, among others (Fig. 5a). The protein-protein interaction (PPI) based on the MCODE algorithm identified five functional clusters shown in Fig. 5b. These functional clusters are formed by networks of proteins related to “extracellular matrix organization”, “muscle contraction”, “cell proliferation”, “cellular oxidant detoxification”, and “carboxylic acid catabolism”, which highlights the effect of age on the vocal fold tissue structure and oxidative stress. Protein members of these functional categories include: The top BP enriched terms related to the proteins affected by hydration status include “protein degradation”, “muscle structure development and system process”, “translation”, “regulation of DNA biosynthesis”, and interestingly, “aging” (Fig. 5c). Five PPI MCODE clusters were identified with the largest network (25 proteins) associated with “protein degradation”, followed by “endocytosis”, “translation”, “muscle structure,” and “extracellular matrix organization” (Fig. 5d). These functional clusters suggest an effect of hydration/dehydration on the vocal fold structure and fundamental cellular processes. Among the top 20 enriched terms for the vocal fold proteins with an interaction effect, “striated muscle contraction”, “positive regulation of sodium ion transmembrane transport”, “extracellular matrix organization”, “tissue homeostasis”, and “aging” are listed (Fig. 5e). The MCODE clusters are associated with “muscle contraction” and “translation” process in the vocal fold tissue (Fig. 5f).

The protein members of the combined enriched GO BP terms forming each functional cluster related to age, hydration, and interaction are listed in Table 1.

**Table 1:** Enriched functional clusters based on MCODE analysis of significant proteins related to age, hydration status, and interaction effect

Functional cluster		Enriched GO BP term*	Protein members (symbols)
1	Age-related	extracellular matrix organization	COL4A2; AIFM2; RAE1; OLA1; COL14A1; TMX3; SLC25A20; DNAJC10; GSTO1; ACACB; EIF4A2; COL4A1; GPX1; IPO5; FBLN5; ENO2; ASPN; SARS1; COL18A1
2		muscle contraction	TPM2; TNNI2; MYL3; MTOR; MYH6; TNS1; MYL2; ILK; DMD; TCAP; DES; NEK9; TNNI1; MYH7; MYL6B
3		cell population proliferation	EIF4B; TXNRD1; ALDH2; XPNPEP1; KYAT3; PGRMC1; H3C6; DDB1; PEPD; CMPK1; MFN1; USP9X; CKB; H3-3B; SQSTM1
4		cellular oxidant detoxification	GSR; MYH10; HSPA2; TXNRD2; MYL4; HADHB; PPIA; RPS27A; TXN; GPX3
5		carboxylic acid catabolism	PEBP1; ECHDC2; HADHA; ACADSB
1	Hydration status-related	proteasome-mediated ubiquitin-dependent protein catabolic process	PSMD6; YWHAB; DDX6; CCT4; PSMD11; HNRNPD; DNAJA2; PFKL; ILK; IPO5; PSMD1; SOD1; PSMC2; TRIP12; PSMC5; CCT8; CCT5; PSMC4; PSMA4; TCP1; PSMD2; HSP90AB1; HSP90AA1; PSMC1; HUWE1
2		endocytosis	LSM2; ME2; DCTN2; UGP2; GOT2; RPL10A; NME1; GNB1; GRB2; USP15; ACTB
3		amide biosynthetic process	EIF4B; EIF4G1; ACSL4; NAA15; SLIRP; NXF1; RCC1; RPL35; ANK3; EIF2S1; GYS1
4		actomyosin structure organization	PDGFRB; TPM1; COL4A2; FBLN5; LAMA2; ASPN; LDB3; PDLIM1
5		extracellular matrix organization	MFAP4; MYL2; MYH2; FBN1; CALU; LAMC1; SVIL; COL18A1; NUP98; LMNA; DMD
1	Interaction-related	muscle contraction	MYL3; FBLN5; NID2; COL18A1; NOS1; TNNI1; MYL2; LAMC1; MYL6B; DMD; LTBP1; MFAP4
2		translation	SNRNP70; RPS15; RPS14; EIF4A2; EIF2A; EIF4B

*the top enriched GO BP (biological process) term of each cluster is used to represent the cluster

### Proteins differentially regulated in the rabbit vocal folds due to age, hydration status, and interaction

**Fig. 6:**
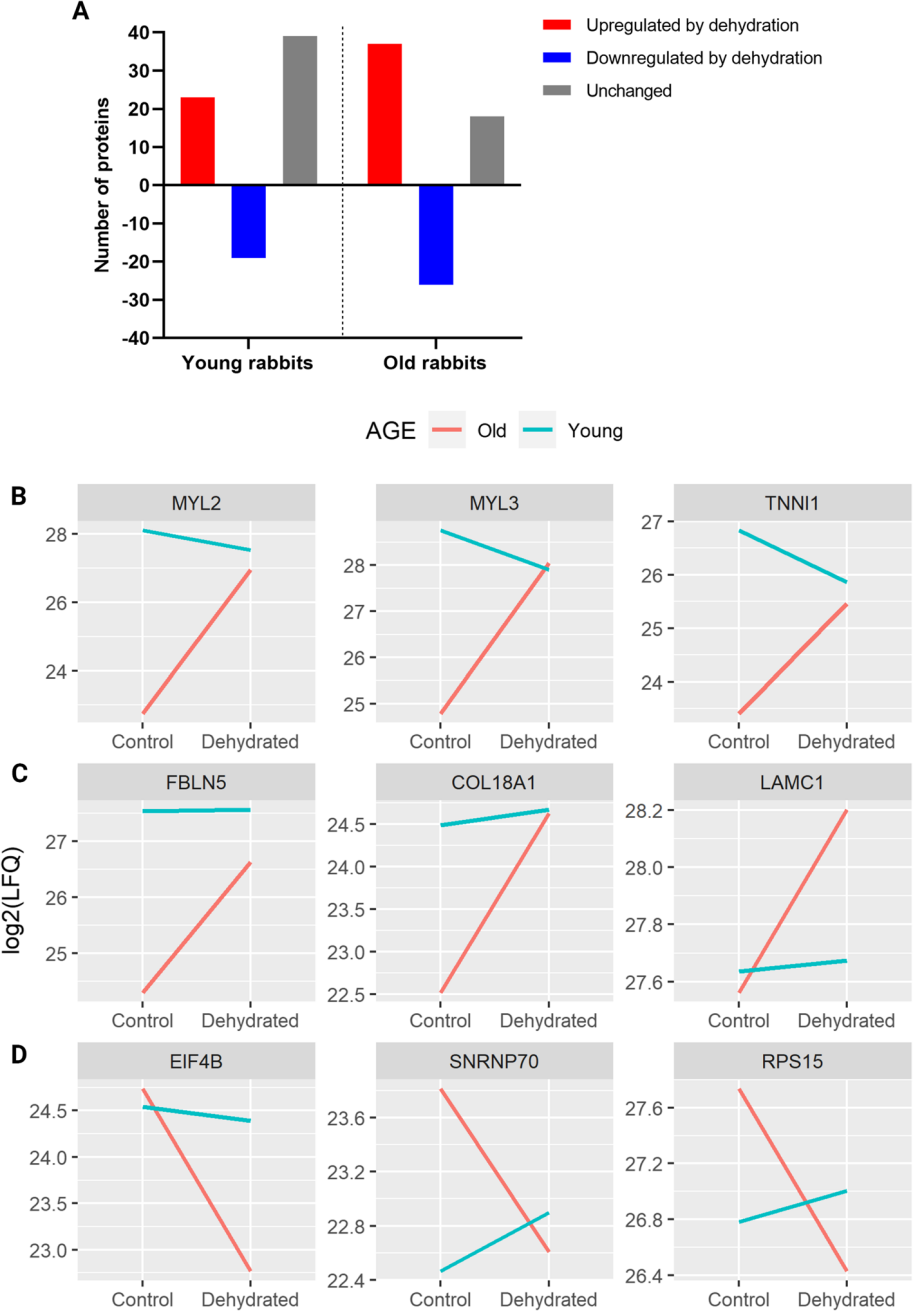
Change in the expression pattern of proteins differentially regulated in the vocal folds of young and older adult rabbits by an interaction effect. **a** Number of upregulated, downregulated and unchanged proteins in the vocal folds of young and older adult rabbits in response to dehydration. Unchanged proteins are proteins with *p* ≤ 0.05 and |Log2(LFQ mean difference)| above the cut-off of 0.38. Plots show the effect of interaction in the pattern of expression for **b** muscle-related proteins, **c** extracellular matrix proteins, and **d** proteins associated with translation in young and older rabbit vocal folds due to dehydration

The age-related protein changes, i.e., proteins differentially regulated by the main effect of age, are associated with downregulation of muscle and extracellular matrix structural proteins in the vocal folds of older adult rabbits compared to young, such as Myosin light chains 2, 3, 4 and 6B, Myosin heavy chains 6, 7 and 10, Troponin I, Fibulin 5, Collagens type XIV and XVIII alpha 1 chain, Serpin H1, and Versican. Desmin, a muscle contraction-related protein, was upregulated in older vocal folds compared to young. Changes related to hydration status resulted in upregulation of proteins involved in muscle function such as Myosin light chain 2 and Tropomyosin alpha-1 chain, and proteins of the extracellular matrix structure including Fibulins 1 and 5, Collagen type XVIII alpha 1 chain, Fibrillin 1, Microfibril associated proteins 4 and 5, in the presence of dehydration. The transcription factor NIF3-like protein 1, with no function previously described in vocal folds, was also upregulated by dehydration. Upregulation of the intermediate filament Lamin A/C, involved in muscle structure development and related to tissue stiffness, was observed as an effect of dehydration, although the magnitude of increase was below the mean difference cut-off used in our study. Among downregulated proteins affected by dehydration, Sarcolemmal membrane-associated protein involved with muscle contraction was identified. The changes in the protein expression patterns related to an interaction effect were analyzed, and the number of proteins upregulated, downregulated, or unchanged (below the cut-off of 1.3-fold change; i.e., |Log2 mean difference| = 0.38) in young and older adult rabbits due to systemic dehydration are shown in Fig. 6a. The fold change cut-off of 1.3 was based on previous studies and considerable changes for large structural proteins [50–52]. Amongst the 81 proteins differentially regulated by an interaction effect, 32 showed an opposite pattern of change in the young and older rabbit vocal folds in response to systemic dehydration, with some proteins considered unchanged in one of the age groups (Additional file 3: Table S3). Seventeen proteins were downregulated in older adults and upregulated in young rabbits, while 15 were upregulated in older adults and downregulated in young rabbits. These opposite change patterns were seen for Myosin light chains 2 and 3, and Troponin I, which were increased by 18.2, 9.6, and 4.1-fold, respectively, in older, and decreased by − 1.5, − 1.8, and − 2-fold, respectively, in young rabbit vocal folds due to dehydration (Fig. 6b). Forty-one proteins presented a change in the expression pattern due to dehydration in only one of the age groups. Thirty-one proteins changed solely in older adult rabbits, while ten changed in the young rabbits alone (Additional file 3: Table S3). Fibulin 5, Collagen type XVIII alpha 1 chain, and Laminin subunit gamma 1 were upregulated by 5, 4.3, and 1.5-fold, respectively, in older adult rabbits due to dehydration, while they remained unchanged in young rabbits (fold change ≤1.3) (Fig. 6c). Proteins involved in translation as the Eukaryotic translation initiation factor 4B and Ribosomal protein S15 were downregulated by − 3.9 and − 2.5-fold, respectively, in older adult rabbits and unchanged in young rabbits, while the Small nuclear ribonucleoprotein U1 was downregulated by − 2.3-fold in older adult and increased by 1.3-fold in young rabbits (Fig. 6d). Interestingly, the overall magnitude of change of differentially regulated proteins was higher in the older adult rabbits compared to young, suggesting that older vocal folds are more susceptible to protein changes caused by systemic dehydration. In addition, a higher number of proteins were considered unchanged (below 1.3-fold change) in the young rabbits compared to 18 in the older rabbits, indicating that more proteins are affected by systemic dehydration in aging vocal folds (Additional file 3: Table S3).

### RT-qPCR of selected genes based on differentially regulated proteins

**Fig. 7:**
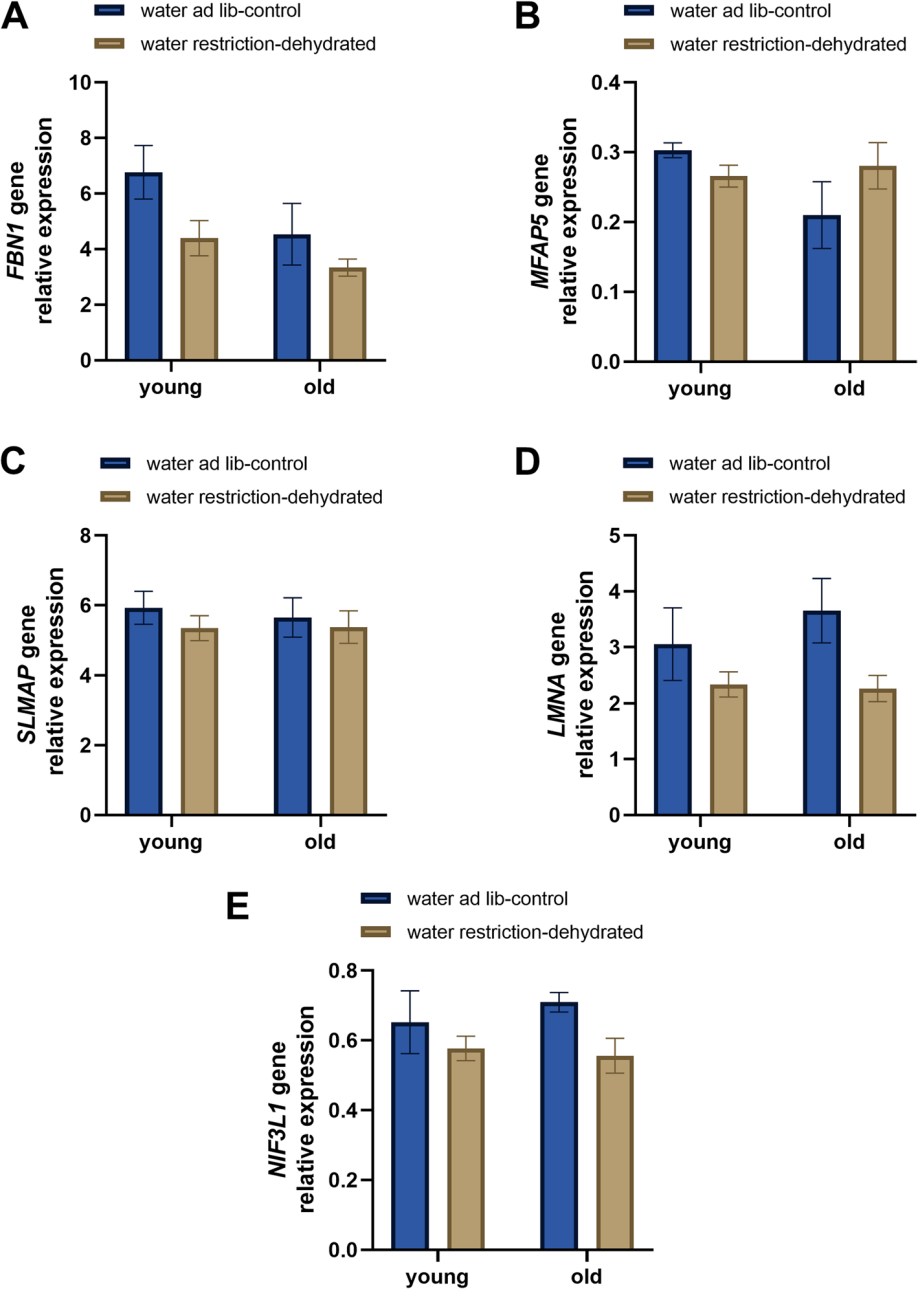
Relative expression of tested genes based on proteins differentially regulated by hydration status. *FBN1*: Fibrillin 1, *MFAP5*: Microfibril associated protein 5, *SLMAP*: Sarcolemmal membrane-associated protein, *LMNA*: Lamin A/C, and *NIF3L1*: NIF3-like protein 1. Bars show relative expression mean ± SEM

**Fig. 8:**
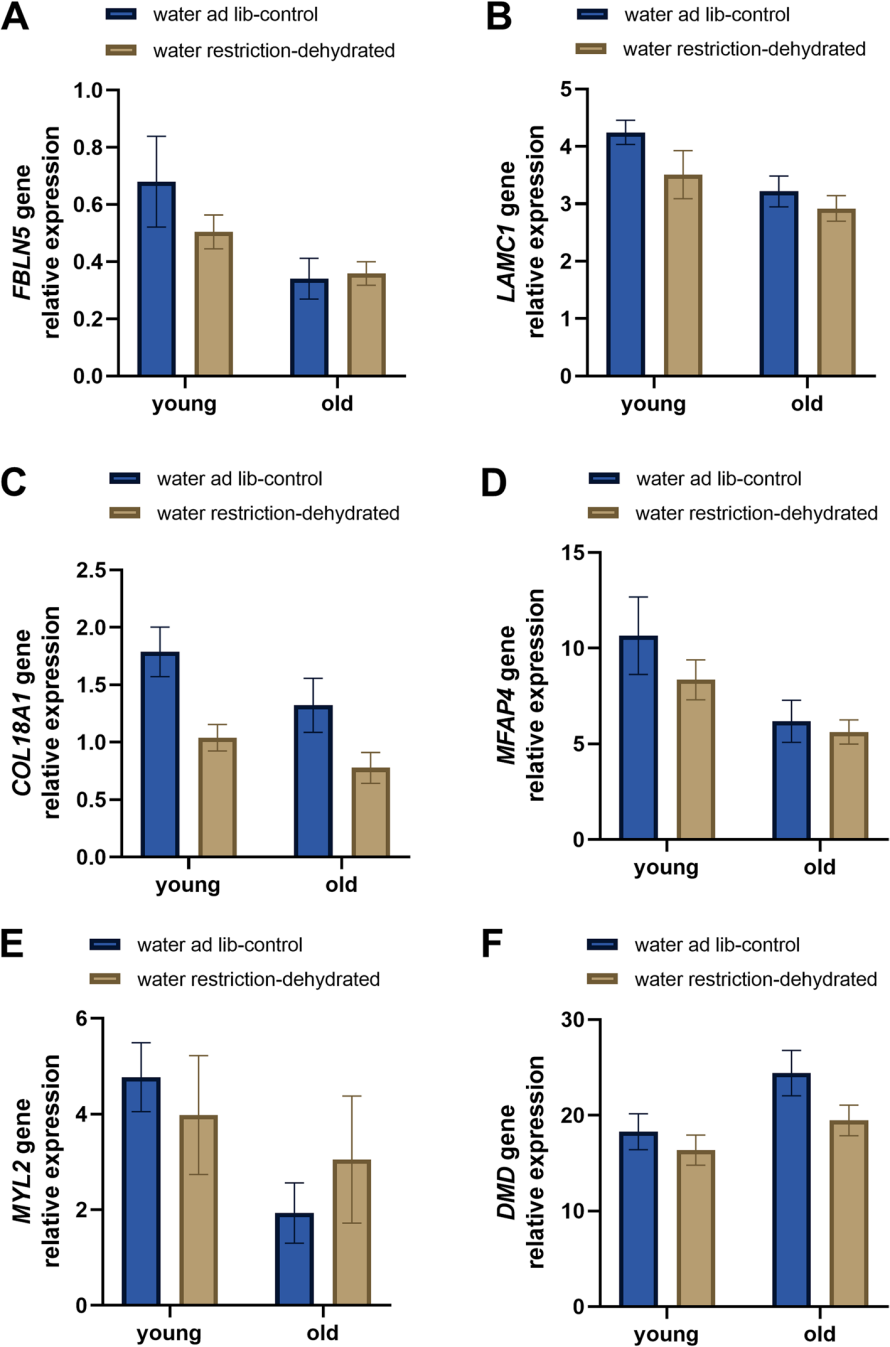
Relative expression of tested genes based on proteins differentially regulated by the interaction of age and hydration status factors. *FBLN5*: Fibulin 5, *LAMC1*: Laminin subunit gamma 1, *COL18A1*: Collagen type XVIII alpha-1chain/Endostatin domain-containing protein, *MFAP4*: Microfibril associated protein 4, *MYL2*: Myosin light chain 2, *DMD*: Dystrophin. Bars show relative expression mean ± SEM

The genes tested by RT-qPCR were chosen based on a subset of proteins identified as differentially regulated by either hydration status or interaction effect. These genes were also selected based on their functional enrichment terms related to extracellular matrix and muscle functions. The transcription factor, *NIF3L1*, involved in neuron differentiation that was upregulated by dehydration, was also tested. Eleven genes were tested; five based on proteins differentially regulated by hydration status (*FBN1, MFAP5, SLMAP, LMNA, NIF3L1*) (Fig. 7a-e), and six selected from the interaction (*FBLN5, LAMC1, COL18A1, MFAP4, MYL2, DMD*) (Fig. 8a-f) results. Interestingly, the expression of *FBN1*, *LMNA,* and *NIF3L1* genes was significantly affected by hydration status (*p* ≤ 0.05) like the respective proteins, but while the proteins were upregulated, the genes were downregulated by systemic dehydration (Fig. 7a, d, and e, respectively). *MFAP5* and *SLMAP* gene expression (Fig. 7b and c, respectively) was not significantly affected by hydration status. *FBLN5* gene was significantly affected by age (*p* ≤ 0.05) and was decreased in older rabbit vocal folds (Fig. 8a), matching the protein expression pattern. *MYL2* gene was not significantly affected by interaction (Fig. 8e) despite having a similar pattern of expression observed for the protein (Fig. 6b). None of the genes tested were significantly affected by interaction, unlike the correspondent proteins.

## Discussion

This study aimed to investigate the proteomic changes in the vocal folds of young and older adult rabbits subjected to the same protocol of water restriction-dehydration: five days of 50% water volume restriction. A water restriction protocol was chosen over pharmacologically-induced dehydration or complete water deprivation to provide a more physiologically-relevant translation to human behavior. A significant proportion of the population of adults and older adults consume about half of the water intake recommended by various governmental institutes worldwide [46–48, 53, 54]. Additionally, we sought to achieve only mild to moderate dehydration to provide further physiological validity to the model. After water restriction, young and older adult rabbits showed body weight losses of − 5.4% and − 4.6%, respectively, consistent with mild to moderate systemic dehydration [55, 56].

LC-MS/MS analysis quantified 2286 proteins in the vocal folds of young and older adult rabbits combined after applying the filtering criteria for this study. These proteins include structural components of the extracellular matrix such as collagens, fibrillin 1 and 2, fibulins, elastins, laminins, proteoglycans, decorin and fibronectin; and muscle proteins such as myosins and actins. These proteins are also present in human vocal folds [57], corroborating our protein extraction and fractionation methods and the LC-MS/MS approach used to analyze the proteome of rabbit vocal folds.

Subsets of proteins were identified as significantly affected by age (young vs. older adult rabbits), hydration status (water ad lib-control vs. water restriction-dehydrated), or interaction of these factors. Interestingly, the majority of proteins affected by the individual factors or the interaction are unique, with only a few common proteins between subsets. These results indicate that the vocal folds have independent mechanisms of protein regulation related to the aging process, response to hydration status, and a combination of age and level of hydration (interaction). Despite the identification of unique proteins regulated by the individual effects considered in this study, the enrichment pathway analysis showed a common association of these proteins to extracellular matrix organization and muscle structure/contraction, suggesting that age and hydration, as single or interacting factors, interfere with the physiological biomechanical and viscoelastic properties of the vocal folds responsible for structural support and voice production [57–62].

Our study revealed several changes related to muscle proteins, especially in older adult rabbits. When comparing the effect of age on vocal fold protein regulation, the downregulation of essential muscle proteins, such as Myosin heavy and light chains and Troponin I, was observed in older adult rabbits. The Myosin heavy chains 6, 7 (β-subunit) and 10, and the Myosin light chains 2, 3, and 4 were downregulated in older rabbit vocal folds, an effect of age alone. Myosin heavy chain β-subunit is expressed in muscle, rich in slow-twitch type 1 fibers; the Myosin light chains downregulated in older rabbit vocal folds are responsible for modulatory and regulatory functions of muscle conformational changes and contraction [63]. Likewise, Troponin I regulates contraction by modulating myofibril’s calcium sensitivity, causing relaxation. The downregulation of these proteins corroborates the presence of muscle decline expected in aged individuals [8]. Studies in humans have shown the decreased synthesis of muscle proteins in middle-aged and older adults compared to their young counterparts [64]. Specifically, Myosin heavy chain decreased with age. Myosin is crucial for muscle contraction by hydrolyzing ATP to generate muscle contraction [63], and its decrease may also contribute to the loss of muscle mass and muscle strength. An opposite interaction effect was observed for Myosin light chains 2, 3, and 6B, which were upregulated in older adult rabbits and downregulated in young rabbits in response to dehydration, suggesting that dehydration also plays a role in the expression of these proteins. There is a direct connection between the effect of hydration/dehydration in muscle cells; water mediates interactions of myosin and actin filaments, thus, interfering with muscle contraction [65–67]. Interestingly, the intermediate muscle filament Desmin was upregulated in older in comparison to young rabbit vocal folds, indicating an effect of aging. Desmin has an important role in cellular homeostasis in response to chronic stress or muscle atrophy [68, 69], and increased Desmin expression has been observed in the muscle of aging rats [70]. However, the impact of these changes have yet to be investigated in the context of the vocal folds and voice function. Nevertheless, these data provide evidence of age alone and interactions between age and hydration status in the laryngeal or vocal fold muscle.

Various extracellular matrix-related proteins were differentially regulated herein. Extracellular matrix proteins provide structural support, mechanical properties, and strength of tissues, including vocal folds, playing a pivotal role in phonation [62, 71, 72]. Collagens XIV, XVIII, and Fibulin 5 were downregulated in older rabbit vocal folds compared to young tissue. To our knowledge, these specific collagen types have not been investigated in depth in vocal fold tissue; however, studies suggest that the changes in the collagen fiber density and arrangements within the lamina propria may affect phonation [73, 74]. Collagen type IV is exclusive to extracellular matrix basal membranes [75] and is present in the human vocal fold basal membrane providing support to epithelial and endothelial cells [76]. Collagen type IV was upregulated in older rabbit vocal folds compared to young, an effect of aging observed in our study. The relationship between Collagen type IV and aging is not well established. Increased accumulation of Collagen type IV is reported in the basal lamina of cerebral microvessels in humans [77] but decreased in the skin of older adults [78]. Conversely, several extracellular matrix proteins were upregulated, including Collagen type XVIII and Fibulin 5, in the presence of dehydration when observing the effect of hydration status alone. These protein changes may be related to the remodeling of the extracellular matrix [79] in response to dehydration. Moreover, the accumulation of collagens and the decrease of elastins may result in extracellular matrix stiffness in aging larynx and other organs [59, 79]. Finally, Lamin A was upregulated by dehydration, by a smaller magnitude, especially when observing the mean difference within the young groups. Previous data has identified that Lamin proteins A and C are important for imparting the nucleus with its stiffness, and their expression has been reported to scale with tissue stiffness [80]. Thus, upregulation of this protein due to dehydration may be related to tissue stiffness in the vocal fold of rabbits.

We tested the relative expression of a small subset of genes based on the proteins differentially regulated in the vocal folds. Four out of the 11 (36%) tested genes followed the direction of the expression pattern of the respective proteins; however, their relative expression was not statistically significant as their proteins. The correlation between gene and protein expression can be as little as 40% depending on the system being investigated [81]. Multiple factors contribute to the inconsistency between mRNA and protein abundance for a given gene, such as mRNA and protein stability, lower rate of mRNA transcription compared to protein translation, and post-translational modifications [81, 82]. Ideally, comparing the complete proteome to the transcriptome is a more desirable approach when the study design and biological replicates allow it.

The number of proteins identified in this study represents an increment of 602 vocal fold proteins compared to our previous study using 6-month-old rabbits (young) subjected to systemic dehydration with furosemide, which identified 1684 proteins [30]. This difference can be due to the different number of biological replicates used in each study, eight rabbits in the furosemide study versus 22 rabbits in the present study, and the filtering criteria applied in each study. Regardless of the differences between studies, 1307 proteins were detected in both, including main extracellular matrix and muscle components and cellular metabolism related-proteins. While this study identified a higher number of vocal fold proteins compared to our previous study [30], the number of differentially regulated proteins due to age, dehydration, or interaction was still relatively low. This might suggest that many vocal fold proteins are regulated at the post-translational level, and measurement of total proteome may only confer a partial view of the overall changes in vocal fold biology. Therefore, future studies that focus on identifying site-specific changes in protein phosphorylation and other modifications may provide a deeper and complete understanding of the regulation of vocal fold proteome under different conditions such as age or hydration status. Our study shows that proteomic analysis can provide critical information and contribute to our understanding of vocal fold biology under different physiological conditions.

The life expectancy of White New Zealand rabbits may reach ten years with proper care, spaying and neutering, but rabbits in research settings have a lower life expectancy due to stress [83]. The young and older rabbits used herein correspond to sexually mature young adults and retired breeders representing older adults, respectively. Studies including elderly rabbits (> 6 years of age) may provide additional age-related protein expression changes. However, the effects of aging were observed in this study in the older rabbits group. Interestingly, as seen in humans [14, 17], the older rabbits in this study had a lower water consumption than young rabbits when provided with water ad libitum*.* Among the indicators of dehydration, packed cell volume (PCV) and total solids (TS) did not change significantly after water restriction; however, the average percentage change of TS in the dehydrated groups is higher than those for the control groups, but the high variability within groups likely prevents a significant statistical difference. Given the lack of sensitivity of these indicators of dehydration, our group has developed a method to quantify *renin* gene expression in the kidneys [28]. Renin is released by juxtaglomerular cells in the kidney in response to decreased blood pressure, and its transcription is expected to be increased in the case of dehydration due to decreased blood volume [84]. In this study, there was a significant increase in *renin* expression in young rabbits subjected to water restriction compared with same-age rabbits receiving water ad libitum; conversely, there was no significant difference in *renin* expression in older adult rabbits between treatments. Interestingly, the impact of age on the kidneys, including a decrease in renin activity, is well documented in aging humans [13, 16, 85]; these factors contribute to the increased susceptibility to dehydration as we age. Thus, it is possible that renin expression was affected by the age in the older rabbits in this study. Nevertheless, the rabbits in both groups were systemically dehydrated based on imposed water restriction and decreased body weight, which is commonly used as a consistent indicator of dehydration in humans [86–88] and animal models [28, 30, 89–92]. Clinical translation to human subjects is preliminary and awaits further investigation. However, given the similarity in rabbit laryngeal anatomy to humans [34, 35], it is appropriate to posit that systemic dehydration in older speakers, as induced by reduced water intake, may impact the extracellular matrix which scaffolds the vocal folds. This structural impact could negatively prime these tissues for trauma from mechanical vibration. The similarities between older adult rabbits and aging humans regarding decreased water intake and renin expression serve as additional support for using the rabbit as an animal model of dehydration.

## Conclusions

Our study successfully used the rabbit as an in vivo model to investigate the impact of aging and hydration status on the proteome of vocal fold tissue. The proteomic data generated in this study provide a robust resource for the laryngological research community. Our findings suggest changes in the relative abundance of extracellular matrix components and muscle-related proteins in young and older rabbit vocal folds with a higher impact of systemic dehydration on the older tissue. The extracellular matrix is directly related to the viscoelastic properties and vibration of the vocal folds, while the muscle fibers scaffold the tissue and are also involved in phonation. The clinical impact of the magnitude of protein changes observed in the rabbit vocal folds, the translation to human vocal folds, and implications for vocal output await further investigation.

## Methods

### Rabbits and water restriction protocol

Experiments were approved by the Purdue Animal Care and Use Committee (protocol #1606001428) and conducted following the guidelines of the National Institutes of Health Guide for the Care and Use of Laboratory Animals [93]. Twenty-two male New Zealand White rabbits (Envigo, Indianapolis, IN, USA) were used in the study. The rabbits were acquired as two age groups: the young group comprised 6.5-month-old rabbits (n = 11), and the older group comprised rabbits between 3.2 and 3.7 years of age (average = 3.3 years old) (n = 11). The 6.5-month-old rabbits are sexually mature young adult rabbits, and the ~ 3.5-year-old rabbits are retired breeders and represent older adults [94]. Male rabbits were used to avoid the effects of female sex hormones on body fluid regulation that occur in hormonally-cycling humans and rodents [95–97]. Animals were acclimatized for one week prior to the experiment. After acclimation, rabbits from both age groups were randomly assigned to either a water ad libitum-control (n = 5 young, and n = 5 older adults) or water restriction-dehydrated (n = 6 young, and n = 6 older adults) group. The experiment consisted of ten days divided into two periods: the *baseline period,* when all rabbits received water ad libitum and had individual water intake measured daily for 5 days, and the *water restriction period.* Rabbits assigned to the water restriction-dehydrated group received half volume of their baseline-average water intake, and control rabbits had free access to water for 5 days (Fig. 1). Body weights and water intake were recorded daily for all rabbits throughout the experiment. Whole blood was collected from all rabbits via ear vein puncture into heparinized microhematocrit capillary tubes to assess the packed cell volume (PCV) and total solids (TS) on day 5 of *baseline* and day 10 of *water restriction*. All rabbits had unrestricted access to food and hay during the entire study. At the end of the *water restriction period*, rabbits were euthanized using 1.0 mL IV Beuthanasia-D Special (Schering Plough Animal Health Corp., Union, NJ, USA).

### Systemic dehydration markers

Percentage changes in body weight, packed cell volume (PCV), and total solids (TS) were assessed as markers of systemic dehydration: [final value after water restriction dehydration (day 10) – baseline value (day 5)]/baseline value*100. Additionally, the relative expression of *renin* gene in the kidney was compared between water ad lib-control and water restriction-dehydrated rabbits in the young and older adult groups.

### Tissue collection

Following euthanasia, kidney cortex tissue and larynges from all rabbits were collected. The kidney tissue specimens were immediately flash frozen in liquid nitrogen, and stored at − 80 °C until processing. Immediately after collecting the larynx, the vocal fold tissue was microdissected under a dissection microscope and flash frozen as described before [30]. The full thickness of the vocal fold tissue, identified as the soft tissue at the level of the arytenoid cartilages at the transverse level of the glottis, was collected bilaterally (left and right vocal folds). Each bilateral section included both mucosa and thyroarytenoid muscle. The left vocal fold was processed for proteomic analysis and the right vocal fold for RT-qPCR.

### Vocal fold protein extraction and preparation for mass spectrometry

Protein extraction and mass spectrometry analysis were performed at the Purdue Proteomics Facility as previously described [30, 98]. Briefly, one vocal fold specimen per rabbit (n = 22) was thawed on ice and washed twice with 25 mM ammonium bicarbonate (ABC) before centrifugation at 6500 rpm for 90 sec. Next, tissues were lysed with 100 mM ABC and 1.4 mm ceramic beads and homogenized using a Precellys tissue homogenizer (Bertin Technologies, Rockville, MD, USA). Homogenates were transferred to new tubes and protein concentration was measured using the bicinchoninic acid assay (BCA) [99].

To increase proteome coverage, protein fractionation was performed for each sample, and the soluble and insoluble fractions were analyzed independently in the mass spectrometer. This procedure was not applied to define the subcellular localization of proteins. The fractionation protocol is described elsewhere [30]. Briefly, fractionation was performed using 50 μg of proteins per sample across all 22 samples. Samples were centrifuged in an Optima MAX-XP ultracentrifuge (Beckman Coulter, Indianapolis, IN, USA) at 55 K rpm for 40 min, and the supernatant containing the soluble fraction was recovered and mixed with 4 volumes of cold acetone and incubated overnight at − 20 °C for protein precipitation. The pellets containing the insoluble fractions were kept at − 20 °C. Following precipitation, soluble fractions were centrifuged at 13,500 rpm for 15 min at 4 °C and pellets were collected and dried in a vaccum centrifuge. Pellets from both soluble and insoluble fractions were incubated with 10 mM dithiothreitol (DTT) and 8 M urea in 25 mM ABC for 1 h at 37 °C for reduction, and 2% iodoethanol, 0.5% triethylphosphine in acetonitrile (ACN) for 1 h at 37 °C for alkylation. Next, samples were dried in a vacuum centrifuge.

Proteins in each sample (22 soluble and 22 insoluble) were digested with trypsin and Lys-C mix (Promega, Madison, WI, USA) at a 1:25 enzyme to substrate ratio, at high pressure using a NEP2320 Barocycler (Pressure Biosciences, South Easton, MA, USA) at 50 °C with 60 cycles of 50 seconds at 20 kpsi, and 10 seconds at atmospheric pressure. MicroSpin C18 silica columns (The Nest Group, Inc., Southborough, MA, USA) were used to desalt the digested peptides and elute with 80% ACN and 0.1% formic acid (FA). Purified peptides were dried in a vacuum centrifuge and stored at − 80 °C until LC-MS/MS. Both soluble and insoluble peptides were resuspended in 3% ACN, 0.1% FA in water at a final concentration of 1 μg/μL, and 1 μL was loaded to the HPLC system.

### LC-MS/MS data analysis

LC-MS/MS data were collected at the Purdue Proteomics Facility. Peptides were analyzed in a Dionex UltiMate™ 3000 RSLCnano System (Thermo Fisher Scientific, Waltham, MA, USA) coupled online to an ETD-enabled Orbitrap Fusion Lumos Mass Spectrometer (Thermo Fisher Scientific) as previously described [30, 98]. Briefly, reverse phase peptide separation was accomplished using a trap column (300 μm ID × 5 mm) packed with 5 μm 100 Å PepMap C18 medium coupled to a 50-cm long × 75 μm inner diameter analytical column packed with 2 μm 100 Å PepMap C18 silica (Thermo Fisher Scientific). The column temperature was maintained at 50 °C. Samples were loaded to the trap column in a loading buffer (2% ACN, 0.1% FA) at a flow rate of 5 μL/min for 5 min and eluted from the analytical column at a flow rate of 150 nL/min using a 280-min LC gradient as follows: linear gradient of 8 to 30% of solvent B in 225 min, 30–60% of B in next 35 min, before reverting back to 2% of B, and hold at 2% of B for next 15 min for column equilibration. The column was further washed and equilibrated by using three 30-min LC gradients before injecting the following sample. All data were acquired in the Orbitrap Fusion Lumos Mass Spectrometer (Thermo Fisher Scientific). Orbitrap mass analyzer and data were collected using a higher-energy collisional dissociation (HCD) fragmentation scheme. For MS scans, the scan range was from 375 to 1500 *m*/*z* at a resolution of 120,000, the automatic gain control (AGC) target was set at 4 × 10^5^, maximum injection time 50 ms, dynamic exclusion 60s, and intensity threshold 5.0 × 10^3^. MS data were acquired in data-dependent mode with a cycle time of 3 s/scan. MS/MS data were collected at a resolution of 7500. The mass spectrometry data underlying this study were deposited in the Center for Computational Mass Spectrometry database under MassIVE ID: MSV000089683 (https://massive.ucsd.edu/ProteoSAFe/static/massive.jsp).

LC-MS/MS data were analyzed using MaxQuant software (version 1.6.3.3) [100] against rabbit (*Oryctolagus cuniculus*) database downloaded from the UniProt (www.uniprot.org) containing 41,458 protein sequences. Soluble and insoluble fractions acquired data were merged for each correspondent sample before database searches. The following parameters were edited for the searches: precursor mass tolerance of 10 ppm; enzyme specificity of trypsin/Lys-C enzyme allowing up to 2 missed cleavages; oxidation of methionine (M) as a variable modification and iodoethanol (C) as a fixed modification. False discovery rate (FDR) of peptide spectral match (PSM) and protein identification was set to 1%. The unique plus razor peptides (non-redundant, non-unique peptides assigned to the protein group with most other peptides) were used for peptide quantitation. Only proteins detected with at least one unique peptide and MS/MS ≥ 2 (spectral counts) were considered valid identifications. Label-free quantitation (LFQ) intensity values were used for relative protein abundance comparisons.

### Statistical analysis for comparative proteomics

Perseus software platform (v1.6.12.0) [101] was used to identify vocal fold proteins differentially regulated between the four groups: young-control (YC), young-dehydrated (YD), older-control (OC), and older-dehydrated (OD). First, MaxQuant data was uploaded to Perseus and “contaminants”, “reverse”, and “only identified by site” proteins were removed. Next, LFQ values were Log2 transformed, and data was filtered to have a minimum of three LFQ valid values (non-zero LFQ values in at least three replicates) in at least one group. After filtering, imputation for missing values was performed sample-wise based on normal distribution. Statistical analysis was completed with Two-way ANOVA to compare the LFQ means of each protein between groups for each given factor and interaction. Significant results were considered for the main effect of age (young vs. older adults; regardless of hydration status), the main effect of hydration status (control vs. dehydration; regardless of age), and the interaction of both factors. Proteins with a *p*-value ≤0.05 were considered significant between age groups, hydration status or interaction. Proteins with a |Log2(LFQ mean difference)| ≥ 0.38 were considered differentially regulated [30, 50].

### Principal component analysis

Principal component analysis (PCA) was completed for each set of Two-way ANOVA results based on age, hydration status, and interaction. The three sets share the same list of proteins, but with different *p*-values depending on the main effect or interaction analyzed (Additional file 1: Table S1). Common proteins between age and interaction, and between hydration status and interaction were removed before the PCA since an interaction effect precludes interpretation of the main effects directly. Proteins in each list were organized by ascending *p*-value and PCA plots were created based on the top 500 proteins (for age and hydration status), top 250 proteins (for interaction), and all significant proteins (*p* ≤ 0.05) for each main effect and interaction combined. Correlations between proteins with *p* ≤ 0.05 for age and hydration status with principal component 1 (PC1) were calculated. PCA and correlation analysis were completed with R (v4.1.2) using RStudioTM version 1.4.1717 (RStudio, PBC, Boston, MA, http://www.rstudio.com).

### Pathway and process enrichment analysis of significant proteins

Three enrichment analyses were performed for the protein subsets identified in the vocal folds as significantly affected by age, hydration status, or interaction of both. Uniprot IDs from these significant subsets of proteins were converted into gene symbols using the UniProt Retrieve/ID mapping tool (https://www.uniprot.org/uploadlists/), which were used as the input for the enrichment analysis with Metascape [49] (https://www.metascape.org). For each given subset of genes, pathway and process enrichment analysis was conducted selecting GO Biological Processes as the ontology source. Enrichment analysis default parameters were applied: minimum overlap of 3 gene counts, *p*-value cut-off of 0.01, and enrichment factor > 1.5 (ratio between the observed counts and the counts expected by chance). All genes in the genome were used as the enrichment background. Enriched ontology terms are grouped into clusters based on membership similarities with the most statistically significant term within a cluster representing that cluster. Metascape calculates *p*-values based on the cumulative hypergeometric distribution [102] and *q*-values using the Benjamini-Hochberg procedure to account for multiple testing [103]. Kappa scores [104] are used as the similarity metric for hierarchical clustering of the enriched terms, and clusters are determined based on sub-trees with a similarity > 0.3.

### Protein-protein interaction enrichment analysis based on significant proteins

Protein-protein interaction (PPI) enrichment analysis was carried out for each subset of genes affected by age, hydration status, or interaction by applying the databases STRING [105], BioGrid [106], OmniPath [107], InWeb_IM [108]. Protein networks were created and the Molecular Complex Detection (MCODE) algorithm [109] was applied to identify densely connected network components and associated functional descriptions. Pathway and process enrichment analysis was applied to each MCODE component independently, and the three best-scoring terms by *p*-value were retained as the functional description of the corresponding components, which are shown along with networks as a color-coded list.

### RNA extraction and RT-qPCR

Total RNA was extracted from renal cortex (*n* = 22) and vocal fold tissue (n = 22; one vocal fold per rabbit) using RNeasy Plus Mini Kit and RNeasy Fibrous Tissue Mini Kit (QIAGEN, Hilden, Germany) following manufacturer’s protocol, respectively. Concentration and purity of RNA was assessed by spectrophotometry (NanoDrop™, Thermo Fisher Scientific). The cDNA synthesis was performed with 1.0 μg and 250 ng of total RNA extracted from renal cortex and vocal fold tissue, respectively, using SuperScript™ IV VILO™ Master Mix (Invitrogen™ by Thermo Fisher Scientific). Target vocal fold genes were selected based on proteins differentially regulated identified in the proteome associated with gene ontology enrichment terms (based on the enrichment analysis described above) related to extracellular matrix and muscle functions. The primers for qPCR were designed using Primer-BLAST tool [110], and at least one primer of each pair span an exon-exon junction to prevent residual genomic DNA amplification (Table 2). Each sample was run in triplicates for each gene in 96-well PCR plates with Power SYBR® Green PCR Master Mix (Applied Biosystems™ by Thermo Fisher Scientific), and a final concentration of 100 nM of each primer per reaction. Reactions were carried out in a QuantStudio™ 3 Real-Time PCR System (Applied Biosystems™) with the thermal cycling conditions of 95 °C for 10 min; 40 cycles of 95 °C for 15 sec, and 60 °C for 1 min; and melt curve stage of 95 °C for 15 sec, 60 °C for 1 min, 95 °C for 1 sec. The relative expression level of kidney *renin* was analyzed with the 2^−ΔΔCT^ method [111] using glyceraldehyde-3-phosphate dehydrogenase (*GAPDH*) as normalizer, and the relative expression of the vocal fold target genes was calculated with 2^−ΔCT^ method [112] using hypoxanthine phosphoribosyl-transferase 1 (*HPRT1*) as normalizer.

**Table 2:** RT-qPCR primers used to test the expression of kidney *renin* as a dehydration marker and vocal fold genes selected based on differentially regulated proteins

Gene symbol	Protein name	mRNA NCBI Ref Seq	Primers 5′ - 3’		Product size (bp)
**Kidney genes**					
*REN*	Renin	XM_017347610.1	Fw	GACATAGTGACTGTGGGCGG	87
			Rv	CTTGGCCAGCATGAAGGGTA	
GAPDH*	Glyceraldehyde-3-phosphate dehydrogenase	NM_001082253.1	Fw	GTCGGAGTGAACGGATTTGG	81
			Rv	GGCGACAACATCCACTTTGC	
**Vocal fold genes**					
*LAMC1*	Laminin subunit gamma 1	XM_017339901.1	Fw	CCTTCGGAGACGAGGTGTTC	95
			Rv	GTTGCACTTGCACCTGCCG	
*FBLN5*	Fibulin 5	XM_002719596.3	Fw	GCTCTCTGCTCCCAACTACC	101
			Rv	CACACTCGTCCACATCCACA	
*COL18A1*	Collagen XVIII alpha-1chain/ Endostatin domain-containing protein	XM_008253234.2	Fw	GGTCAACCTCAAGGACGAGG	198
			Rv	GTCTCGCAGTAGCTGTTGGT	
*MFAP4*	Microfibril associated protein 4	XM_002722656.3	Fw	GGCAAGTGGACGGTTTTCC	71
			Rv	TTGTAGTCATTCCAGCCCCG	
*FBN1*	Fibrillin 1	XM_008269057.2	Fw	TCAATGGCTACCCCAAACGG	78
			Rv	CAGACTGATCCTCAACAGTGG	
*MFAP5*	Microfibril associated protein 5	XM_002712855.3	Fw	CAGGGAGTGGCTTTGTTCCT	182
			Rv	CCAAGCGCATCCATTCAGC	
*MYL2*	Myosin regulatory light chain 2	XM_002719750.3	Fw	GGGAGAAACTCAAGGGAGCG	95
			Rv	GTAGTCGGCCTTCAGCACTC	
*DMD*	Dystrophin	XM_017349731.1	Fw	TGCGGTTCAGAAGCAAAACG	170
			Rv	TCTCATCAGGAGGCAGCTCT	
*SLMAP*	Sarcolemmal membrane-associated protein	NM_001082348.2	Fw	GCACAGTCAGAAACTGAGGC	196
			Rv	TCTGCAGTTGGGCTTGAAGA	
*LMNA*	Lamin A/C	XM_008264325.1	Fw	CAAGACCCTCGACTCTGTGG	102
			Rv	TTCTTGGTGTTGCGCGCTTT	
*NIF3L1*	NIF3-like protein 1	XM_002712443.3	Fw	TCGGAGCCTGTACATCCAGA	180
			Rv	CCTCACCATCAGTCCTAGCAG	
HPRT1*	Hypoxanthine phosphoribosyltransferase 1	NM_001105671.1	Fw	GATGGTCAAGGTCGCAAGCC	73
			Rv	TCCAACAAAGTCTGGCCTGT	

*normalizer genes used for relative expression calculation of target genes

### Statistical analysis

The data for baseline water intake, % changes in body weight, PCV and TS, and kidney *renin* gene expression were tested for normal distribution with Shapiro-Wilk test (α = 0.05). For comparisons between two groups, unpaired t-test was applied when data passed the normality test, and Mann-Whitney test, when data did not pass normality. Two-way ANOVA was applied for comparisons with two factors, such as water intake between the *baseline* and *water restriction period* (Two-way repeated-measures ANOVA followed by Sidak’s test), and relative gene expression of vocal fold genes between rabbits (ordinary Two-way ANOVA). GraphPad Prism 9.3.1 software was used for the statistical analyses and graphics. Significance was defined at α = 0.05.

## Supplementary Information

**Additional file 1.**

**Additional file 2.**

**Additional file 3.**

## Data Availability

The proteomic data generated and analyzed during the current study are available in the MassIVE repository under ID MSV000089683: https://massive.ucsd.edu/ProteoSAFe/dataset.jsp?task=fff675b02b354a669eb130c4d6923962
